# Phytase Improves Zinc Utilization by Broiler Chickens

**DOI:** 10.3390/ani14233423

**Published:** 2024-11-27

**Authors:** Douglas Drebes Brunhaus Maria, Sergio Luiz Vieira, Raquel Medeiros Horn, Maria Luísa Adachi Marchi, Andre Favero

**Affiliations:** Department of Animal Science, Federal University of Rio Grande do Sul, Av. Bento Gonçalves, 7712, Porto Alegre 91540-000, RS, Brazil; douglas.drebes@gmail.com (D.D.B.M.); rakihorn@gmail.com (R.M.H.); marialuisaadachi@gmail.com (M.L.A.M.); afaverors@gmail.com (A.F.)

**Keywords:** broiler, phytase, zinc

## Abstract

The study aimed to evaluate phytase effects on the availability of zinc (Zn) from corn and soybean meal feeds for broiler chickens, whereas, in parallel, Zn requirements were investigated. A total of 640 Cobb × Cobb 500 male chicks were fed a Zn-deficient diet (18.87 ± 0.87 mg/kg Zn) until 7 days of age. The experiment was a 2 × 5 factorial with 10 treatments (feeds with or without phytase × 5 increasing dietary Zn levels) with 8 replications of 8 birds each. Supplemental Zn sulfate heptahydrate was used (measurements of the Zn analyzed in the feeds were 30.1 ± 0.73, 61.6 ± 0.13, 90.4 ± 1.60, 123.6 ± 1.99, and 151.9 ± 1.84 mg/kg, respectively). There were no interactions between phytase and Zn for any evaluated response. Phytase improved broiler performance and increased plasma myo-inositol, Zn content in the liver and tibia, Zn retention, and digestible energy (*p* < 0.05). No significant impact of dietary Zn was found on broiler performance (*p* > 0.05) except for Zn excretion and tibia Zn content. Adding phytase at 4000 FYT increased the apparent ileal Zn digestibility by 98% and Zn retention by 13.7%, whereas Zn supplementation of up to 151.9 mg/kg did not impact broiler performance.

## Simple Summary

Zinc (Zn) is an essential mineral for animal growth, performing a wide variety of biological functions. It is absorbed in the small intestine and stored as metallothionein, the synthesis of which depends on dietary Zn contents. In poultry feeds, ingredients such as corn and soybean meal (SBM) contain Zn, but its availability for broilers is reduced due to its chelation with phytic acid. The enzyme phytase has been widely added into feeds, potentially increasing the availability of minerals. Zinc content of plant-feed ingredients is usually not considered as a source of this mineral when feed formulating, and this can lead to deficiencies or excesses due to inaccurate supplementation. As it has been shown for other positively charged trace minerals, the potential release of Zn from plant feedstuffs by phytase may reduce the need for its supplementation, aiming for a lower excretion of Zn into the environment.

## 1. Introduction

Zinc (Zn) is essential for broiler growth, with its organic demands including more than 300 enzymes [1]. Since the demonstration of a Zn requirement for the growth of *Aspergillus niger* [2], Zn has shown to be essential for other microorganisms as well as plants and animals [3]. After Fe, Zn has been previously shown to be the second most abundant trace metal in the human body, having the widest variety of functions when compared to any other trace element [4].

Dietary Zn has to be in a soluble form in order to be absorbed, which occurs in the upper small intestine in a saturable carrier-mediated process [5,6]; however, a non-saturable mechanism also exists at the ileum of chickens [7]. Once absorbed, Zn is predominantly stored as metallothionein (MT), a low-molecular-weight protein having selective capacities to bind metals, and thus serving as a major storage form of trace elements, such as Zn and Cu as well as Cd, Hg, and Ag [8]. The genetic expression of MT is Zn dependent [9] and, therefore, one can expect its increase by increasing dietary Zn.

Corn and soybean meal (SBM) have considerable Zn content [10], which, however, seems to be of variable availability for poultry [11]. One of the factors affecting Zn utilization from plant feedstuffs is its chelation with phytic acid [12], which form an insoluble crystal at intestinal pH [13]. Phytate content ranges from 0.21% in corn to 0.37% in SBM [14,15], binding from 1 to 3% of its molecular weight as P, plus considerable amounts of other positively charged minerals [13]. Phytase addition to broiler feeds has become widespread as an affordable strategy when minimum cost linear feed formulation is used because of its favorable effects on P and Ca availability [16]. Phytases also increase the availability of trace minerals, such as Fe [17] and Cu [18].

It has been earlier demonstrated that broilers require a minimum of 55 mg/kg total dietary Zn to avoid deficiency signals such as retarded growth and enlarged hocks [19]. The most recent suggestions for Zn supplementation in feeds for the presently commercially grown broilers vary from 65 to 120 mg/kg [15,20,21,22]. These recommendations do not take into consideration the Zn contents in corn and SBM as well as in other plant feedstuffs. Such important differences in dietary supplemental recommendations indicate the uncertainties surrounding Zn availability in poultry feeds. Therefore, deficiencies or excesses of Zn may occur, which, in both cases, lead to economic losses. Factors impacting Zn utilization may differently affect the need for growth in comparison to other metabolic responses, such as enzyme expression.

Concerns about excessive mineral excretion and increased global scale environmental pollution have led to the establishment of a maximum content of 100 mg/kg of Zn in poultry feeds [23]. This may not be a comfortable limit when the wide, related needs of Zn to optimize broiler meat production are confronted with the lack of knowledge on Zn contents and its availability in different feed ingredients.

The hypothesis is that phytase supplementation in broiler feeds may increase Zn bioavailability by releasing phytic acid-chelated Zn into SBM feed ingredients, thereby reducing the high levels of Zn supplementation. This may minimize Zn excretion into the environment without compromising growth in broilers. The present study aimed to evaluate Zn requirements of broiler chickens in the presence of phytase. Zinc sulfate was added in graded increases so that different broiler responses could be assessed. Phytase was added in excess of its commercial recommendation so that evaluations could be done with an expected maximum phytate degradation.

## 2. Materials and Methods

### 2.1. Bird Husbandry and Dietary Treatments

A total of 640 1-d-old male Cobb × Cobb 500 chicks were randomly placed into 80 wire cages (0.9 × 0.4 m^2^). Each cage was equipped with one trough feeder and one nipple drinker, both of inox steel, allowing ad libitum access to water and mash feeds. The temperature at placement was 32 °C, which was adjusted weekly to maintain bird comfort. Lighting was provided 24 h continuously throughout the first week, followed by a 16 h light–8 h dark periods toward the end. Chicks were given a common Zn-deficient diet (18.87 ± 0.87 mg/kg Zn) formulated with corn, polished white rice, and soybean protein concentrate (60% crude protein) from 1 to 7 d. Starting on day 8, cages of chicks were completely randomly allocated into the dietary treatments, which were a 2 × 5 factorial (with or without phytase and five graded increases of supplemental Zn) until 28 d. Each treatment was replicated 8 times; therefore, there were a total of 10 treatments with 8 replications of 8 birds each. Feeds were formulated with energy and nutrients to provide growth comparable to that obtained in commercial settings (2975 kcal/kg AME with 23% crude protein (CP), 1.1% Ca, and 0.50% non-phytate P (nPP) in the first week and 3000 kcal kg/kg ME with 21.6% CP, 1.0% Ca, and 0.48% nPP from 8 to 28 d). Trace mineral supplementation, void of Zn, was commonly provided to all treatments (Table 1).

The commercial phytase was a commercially available product (Ronozyme HiPhorius, 40,000 FYT/g, Novozymes A/S, Bagsvaerd, Denmark). This was added at 100 g per metric ton to deliver 4000 FYT/kg (4150 ± 320 analyzed). The addition of phytase was in excess of its commercial recommendation of 1000 FYT and done to overwhelmingly and quickly degrade the phytate present in corn and SBM, therefore maximizing the release of Zn. Phytase was added into the feeds without attributing value for P and Ca to avoid confounding effects on overall performance. Supplemental Zn was from laboratory-grade Zn sulfate heptahydrate (ZnSO_4_·7H_2_O), which was added to the experimental feeds at 0, 30, 60, 90, and 120 mg/kg (Sigma Aldrich, St. Louis, MO, USA). Calcium carbonate and phosphoric acid used in feeds were also laboratory grade and labeled without Zn contents. Analyses performed in the water demonstrated that the birds did not present detectable Zn. Celite (Celite, Celite Corp., Lompoc, CA, USA) was added as an indigestible marker, at 1% of all experimental feeds, which had an average geometric diameter of 1.109 μm ± 1.26. Phytase and Zn were added to the feeds in 1 kg mixes diluted with SBM.

### 2.2. Growth Performance, Total Excreta, Ileal Contents

Body-weight gain (BWG), feed intake (FI), and feed conversion ratio (FCR) corrected for the weight of dead birds were evaluated at 8, 14, 21, and 28 d. Excreta were collected twice daily on wax paper from 21 to 24 d, and then immediately mixed and pooled by the cage and stored at −20 °C until analysis. Ileal contents were collected from all birds at 28 d, following euthanasia by electrical stunning at 45 V for 3 s from a section of intestine from Meckel’s diverticulum to approximately 2 cm cranial to the ileum cecum junction. Contents were flushed with distilled water into plastic containers, pooled by cage, immediately frozen in liquid nitrogen, and stored in a freezer at −20 °C until lyophilized (Christ Alpha 2-4 LD Freeze Dryer, Newtown, UK). Feeds and freeze-dried samples of ileal contents were ground to pass a 0.5 mm grinder screen (Tecnal, TE-631/2, São Paulo, Brazil).

### 2.3. Analyses and Calculations

Phytase activity in the feeds was analyzed and expressed in phytase yield units (FYT, defined as the activity that releases 1 μmol of inorganic phosphate from 5.0 mM sodium phytate/min at pH 5.5 and 37 °C) [24]. Dry matter (DM) analysis of all samples was performed after oven drying at 105 °C for 12 h [25]. Ileal contents, excreta, and feed samples were analyzed for gross energy (GE) using a calorimeter calibrated with benzoic acid as a standard (IKA Werke, Parr Instruments, Staufen, Germany). Crude protein (N × 6.25) was determined by the combustion method [25]. Acid insoluble ash, ileal samples, and excreta were determined according to the literature [26,27]. Apparent ileal digestibility, total tract retention, IDE, and AME were calculated following the equations of Kong and Adeola [28]. Zinc was analyzed by induction coupled plasma (5800 ICP-OES, Agilent Technologies, Santa Clara, CA, USA).

### 2.4. Blood and Tissue Sampling

Blood samples were taken by heart punctures prior to slaughter from 3 broilers randomly selected from each cage prior to stunning. Obtained blood was partially transferred into 0.5 mL test tubes containing EDTA for further analysis of myo-inositol (MYO) and CuZnSOD. Myo-inositol concentration in plasma was conducted by mass spectrometry, using the UPLC^®^ system (Acquity UPLC^®^ System, Waters, Milford, CT, USA), following the method of Leung et al. [29]. Plasma CuZnSOD was measured as suggested by Gao et al. [30]. Liver samples were collected for analysis of MT and Zn content at 28 d. Collected samples were stored in plastic bags by cage and remained at −20 °C until analysis, and analyses of its concentration were done in one liver randomly taken per replication at 7 and 28 d using the Chicken Metallothionein ELISA Kit (Ref. MBS260362, MyBioSource, San Diego, CA, USA, EUA). Livers from the other 3 chickens were also randomly taken and later submitted to ethyl-ether extraction following previous acid hydrolysis with hydrochloric acid [31]. Samples were further ashed and Zn content was determined as done with the feeds. The left tibiae of all birds from each cage were collected and had the surrounding muscle tissue removed. Tibia morphometry was done using a micrometer (Model IP65, Mitutoyo Corp., Kawasaki, Japan) in the caudal zone and cranial region, with these values being averaged for statistical analysis. Tibiae were then defatted and ashed to check for Zn content.

### 2.5. Statistical Analysis

Data were tested for homoscedasticity and normality of the variance [32,33]. Normally distributed data were submitted to a two-way ANOVA (phytase and dietary Zn). Analyses were done using the GLM procedure of SAS [34], with significance accepted at *p* ≤ 0.05. Mean separation was done using the Tukey multiple-range test when the model effect was significant [35]. Regression analyses were conducted for the effects of dietary Zn using linear (L) and quadratic polynomial (QP) models.

## 3. Results

The analyzed phytase were 4052 ± 232, 4130 ± 240, 4213 ± 184, 4319 ± 145, 4013 ± 235 FYT/kg in feeds having Zn sulfate added from 30.1 to 151.9 mg/kg in 30 mg/kg increments (The Zn analyzed were: 30.1 ± 0.73, 61.6 ± 0.13, 90.4 ± 3.60, 123.6 ± 1.99, and 151.9 ± 1.84 mg/kg, respectively). No interactions between phytase and dietary Zn were found for any evaluated response throughout the study. Feeds were considered acceptable for the experimental assessment originally planned since analyzed phytase and Zn were in close range with the expected feed formulation (Table 1). There was no mortality, and no individuals were diagnosed as sick during the study.

Phytase supplementation did not affect tibia weight, ash and its morphometry, plasma CuZnSOD activity, or Zn intake (*p* > 0.05). When compared to broilers fed diets without phytase, those having the enzyme added had higher BWG and lower FCR (*p* < 0.05; Table 2), as well as increases in the contents of Zn in the tibia (*p* < 0,05; Table 3) and liver as well as in total MT and plasma MYO (*p* < 0.05, Table 4). Phytase also led to increases in the ileal digestibility of DM, CP, energy, and Zn and, in parallel, Zn retention and AME was increased (*p* < 0.05; Table 5).

Dietary Zn supplementation did not affect broiler performance, tibia weight, ash and morphometry, MT, plasma MYO concentration, apparent ileal digestibility, Zn excretion, DM, or AME (*p* > 0.05).

However, increases in Zn contents in the tibia and liver were quadratically adjusted (Y = 3.771772048 + 0.018993389x − 0.000064899x^2^; R^2^ = 0.2083, *p* < 0.01; Y= 85.43293704 − 0.35548959x − 0.00133237x^2^, R^2^ = 0.07, *p* < 0.05, respectively). In addition, linear reduction was observed in the CuZnSOD activity (Y= 12.33221 − 0.01573x, R^2^ = 0.14, *p* < 0.01), as well as increased linear Zn retention (Y = 0.87236 + 0.17010x, R^2^ = 0.90, *p* < 0.01) and excretion (Y= −0.37247 + 0.12858x, R^2^ = 0.87, *p* < 0.01).

## 4. Discussion

Including phytase in commercial poultry feeds is economically imperative nowadays, with its effects on P and Ca availability being well known. Other benefits of phytase for broilers have been reported, including increases in the availability of amino acids and other minerals [17]. Phytase supplementation, resulting in the increased retention of nutrients, has partially relieved the environmental burden of animal agriculture worldwide [36]. It has been recently reported that broilers fed phytase added to corn-SBM feeds had increases of 8.1 and 10.2% in the availability of Cu and Fe [17,18], respectively. The model utilized in the present study was the same as with Cu and Fe as cited above, which has the advantage of having the trace mineral of interest derived only from corn and SBM. In the present study broilers fed diets having phytase showed higher BWG and lower FCR when compared to those that were not fed the enzyme. Phytase was added in the feeds without attributing any P or Ca values in the formulation; therefore, the performance improvements obtained had other origins. Non-phytate phosphorus (nPP) and the total Ca formulated in feeds used in the present study were typical values found in commercial feeds [21,22].

Information related to Zn availability from plant feedstuffs is variable and, therefore, Zn in commercial feeds is generally supplemented at fixed amounts per ton of feed, regardless of whether it is Zn derived from corn, SBM, or any other ingredient. Chicken excreta is high in Zn [37], which suggests that dietary Zn in commercial feeds is excessive. This was the reason behind the establishment of a maximum allowed Zn content in animal feeds in some countries [23]. On the other hand, Zn is frequently used as a soil amendment in some tropical areas [38]; therefore, the pollutant potential represented by Zn from chicken excreta is obviously dependent on where the chicken excreta are laid up. It is important to note that the continuous increase in the market cost of minerals, including Zn [39], must be considered in the production of sustainable chicken meat. The phytase added in the present study led to an increase of 98% in the apparent ileal digestibility of Zn as well as of 13.7% in the overall retention of Zn.

The feeds used in the present study were formulated with corn and SBM, where limestone and phosphate were replaced by laboratory-grade Ca carbonate and phosphoric acid, both without any detectable Zn content. The mineral premixes utilized did not contain Zn, so any significant variation in dietary Zn resulted from the supplement gradually added. The results of the present study demonstrated that feeds without any Zn supplementation were able to optimize broiler growth from 8 to 28 d, since there were no responses to its dietary increases. No effects of increasing Zn supplementation to 90 mg/kg in phytasesupplemented samples were recently found by Phillippi et al. [40]. Therefore, Zn supplementation in corn-soy feeds seems unnecessary.

Myo-inositol is a cyclic polyalcohol formed by the removal of phosphates from phytate that has metabolic functions similar as those of insulin, including the transport of glucose into cells, especially in muscle and adipose tissue, regulating glucose uptake by the body [41,42,43]. Broilers fed phytase in the present study had a 49.3% increase in plasma MYO concentration, which occurred in parallel to the increases in IDE and AME [44,45,46]. Zinc, on the other hand, has not shown an effect on plasma MYO.

Livers and kidneys serve as a major storage site for the essential trace elements [47,48]. The transcription rates of MT genes increase by the synthesis of more protein to bind the excess Zn and Cu [8]. On the other hand, rapid degradation of MT occurs when Zn is reduced [49]. In the present study, phytase supplementation led to an increase in liver MT content of 11.04% when compared to the non-phytase-supplemented counterpart; however, there was no response for the increases in supplemental Zn.

An important antioxidant enzyme, CuZnSOD, accounts for approximately 90% of total superoxide dismutase activity and protects the tissues against oxidative damage [50]. The structural function and biological activity of CuZnSOD are dependent on the presence of Zn, which is essential for the formation of the enzyme’s active site [51]. In the present study, phytase had no effects on CuZnSOD plasma, whereas increases in dietary Zn at 151.9 mg/kg was linearly correlated with the reduction of the enzyme activity. Studies with Caco-2 cells indicated that high Zn contents affected Cu transport and the efflux from cells by affecting the activity of specific Cu transporters [52]. Additional MT synthesis induced by Zn can negatively impact Cu absorption [53], and this process may lead to the reduction in serum Cu concentrations, directly impacting the activity of the CuZnSOD [54].

Bones are relevant mineral storage sites, and tibia Zn content is an indicator of dietary Zn content [55]. In the present study, increases in tibia Zn occurred both due to the added phytase as well as to increases in dietary Zn. These effects were independent from each other, with phytase leading to an increase of 5.62% in tibia Zn, whereas the quadratic effects of dietary Zn estimated a maximum content of 5.15 mg. Liver Zn contents are indicators of absorbed Zn, which may exceed the needs for a healthy animal metabolism and, therefore, are processed for excretion. In the present study, liver Zn increased 37.1% when phytase was supplemented and, in parallel, a quadratic effect, with a maximum of 109.1 mg/kg Zn, was found when dietary Zn was at 133.4 mg/kg. Previous studies have reported increased Zn in the liver along with its increases in feeds [56]. The proportional increase of liver Zn with the higher intake of the mineral did not induce any sign of toxicity, similarly to what has been observed in previous studies [57].

A significant increase in Zn excretion was observed in the present study, which paralleled body retention as dietary Zn increased, which has been previously shown [55]. The frequent use of broiler excreta as soil amendment plays a significant role in the continuous increase in Zn in areas where this practice is common. It is naturally expected that linear Zn excretion following its dietary intake serves as an indication of excess, especially when considering that no further benefit in broiler growth as well as on other metabolic biomarkers were found [58].

## 5. Conclusions

The inclusion of phytase in broiler feeds was demonstrated to improve the performance of broiler chickens while reducing Zn excreted into the environment. High Zn contents in poultry excreta seem to demonstrate excessive use of Zn in commercial feeds since the gradual increases in supplementation until 151.9 mg/kg did not lead to any benefits on broiler live performance. Overall, the benefits of adding 4000 FYT of phytase were well demonstrated, both in growth parameters and in the availability of Zn from corn and SBM. Benefits of supplementing Zn were not observed in growth, but increased retention indicates possible advantages in the body reserves.

## Figures and Tables

**Table 1:** Ingredient and nutrient composition of feeds having supplemental phytase and graded increases of Zn ^1^.

Ingredient, kg/ton	Depletion (1 to 7 d)	Experimental ^2^ (8 to 28 d)
Corn, 7.8%	12.00	51.54
Soybean meal, 45%	-	37.80
Soybean protein concentrate (60.0% CP)	27.69	-
Polished white rice	54.54	-
Soybean oil	0.41	4.35
Calcium carbonate	2.64	2.42
Phosphoric acid, 85% P	1.49	1.46
Salt	0.51	0.54
DL-Methionine, 99%	0.30	0.32
L-Lysine HCl, 78%	0.04	0.19
L-Threonine, 98.5%	0.06	0.13
Choline chloride, 60%	0.12	0.05
Vitamin and mineral mix ^3^	0.20	0.20
Celite ^4^	-	1.00
Total	100.00	100.00
Calculated nutrient composition, % unless noted		
AMEn, kcal/kg	2975	3000
CP	23.1 (23.2 ± 1.54)	21.6 (21.4 ± 0.38)
Ca	1.10 (1.09 ± 0.15)	1.00 (1.17 ± 0.03)
Available P	0.50	0.48
Total P	0.63 (0.67 ± 0.14)	0.70 (0.69 ± 0.05)
Na	0.23	0.23
Choline, mg/kg	1600	1600
Dig. Lys	1.24	1.22
Dig. TSAA	0.93	0.91
Dig. Thr	0.84	0.82
Dig. Trp	0.27	0.24
Dig.Val	0.93	0.91
Dig. Arg	1.59	1.34
Zn, mg/kg	14.9 (18.9 ± 0.87)	28.6 (30.1 ± 0.73)
Phytase, FYT/kg ^5^	None	4000 (4150 ± 320

^1^ Supplemental Zn was from laboratory-grade ZnSO_4_·7H_2_O (Sigma Al-drich, St. Louis, MO, USA) and added to the experimental feeds at 0, 30, 60, 90, and 120 mg/kg. ^2^ Analyzed Zn measurements were 30.1 ± 0.73, 61.6 ± 0.13, 90.4 ± 1.60, 123.6 ± 1.99, and 151.9 ± 1.84 mg/kg. ^3^ Composition per kilogram of feed: vitamin A, 12,000 IU; vitamin D3, 3000 IU; vitamin C, 50 mg; vitamin E, 100 IU; vitamin K3, 6 mg; vitamin B12, 35 µg; thiamin, 3 mg; riboflavin, 15 mg; vitamin B6, 6 mg; niacin, 40 mg; pantothenic acid, 25 mg; folic acid, 4 mg; biotin, 0.3 mg; Mn, 100 ppm; Cu, 15 ppm; Se, 0.45 mg; and I, 2 mg. ^4^ Indigestible marker (Celite, Celite Corp., Lompoc, CA, USA). ^5^ Ronozyme HiPhorius 40,000 FYT/g (Novozymes A/S, Bagsvaerd, Denmark).

**Table 2:** Growth performance of broilers fed diets with supplemental phytase and graded increases of Zn from 8 to 28 d ^1^.

Phytase, FYT/kg ^3^	BWG ^2^, g				FCR				FI, g			
	8–14	15–21	22–28	8–28	8–14	15–21	22–28	8–28	8–14	15–21	22–28	8–28
None	222	446	611	1279	1.284	1.354	1.460	1.392	285	604	892	1781
4000	241	461	633	1335	1.165	1.305	1.428	1.338	281	602	904	1787
Zn, mg/kg ^4^												
28.6	229	454	623	1306	1.257	1.348	1.431	1.372	288	612	892	1792
58.6	230	453	615	1298	1.204	1.348	1.450	1.371	277	611	892	1780
88.6	231	448	626	1305	1.225	1.325	1.460	1.372	283	594	914	1791
118.6	232	455	624	1311	1.219	1.320	1.448	1.363	283	601	904	1788
148.6	235	456	621	1312	1.212	1.307	1.430	1.348	285	596	888	1769
SEM	1.631	2.398	3.177	5.337	0.009	0.0006	0.005	0.004	1.802	3.044	4.055	6.013
Probability												
Phytase	<0.0001	0.0024	0.0003	<0.0001	<0.0001	<0.0001	0.0023	<0.0001	0.1766	0.7133	0.1185	0.6559
Zinc	0.7699	0.8066	0.7687	0.8623	0.2866	0.0998	0.3025	0.0594	0.3780	0.2145	0.2193	0.7162
Phytase vs. Zinc	0.6860	0.4096	0.0604	0.0664	0.8015	0.5554	0.1957	0.8977	0.1840	0.5400	0.2470	0.1588

^1^ BWG, body weight gain; FCR, feed conversion ratio corrected for the weight of dead birds; FI, feed intake. ^2^ Chick body weight at 1 and 8 d respectively: 44.6 ± 0.99; 148.8 g ± 1.75. ^3^ Ronozyme HiPhorius 40,000 FYT/g (Novozymes A/S, Bagsvaerd, Denmark). ^4^ Formulated at 28.6, 58.6, 88.6, 118.6, and 148.6 mg/kg by adding laboratory-grade ZnSO_4_·7H_2_O; the analyzed values were 30.1 ± 0.73, 61.6 ± 0.13, 90.4 ± 1.60, 123.6 ± 1.99, and 151.9 ± 1.84 mg/kg.

**Table 3:** Tibia morphometry of broiler chickens at 28 days of age fed diets with supplemental phytase and graded increases of Zn from 8 to 28 d.

	Length	Proximal Epiphysis	Diaphysis	Distal Epiphysis	Weight	Ash	Zn ^3^
Phytase, FYT/kg ^1^	cm				g	%	mg/kg
None	7.42	1.95	0.77	1.64	7.00	23.7	4.70
4000	7.46	1.98	0.76	1.66	7.12	23.5	4.98
Zn, mg/kg ^2^							
28.6	7.49	1.99	0.75	1.68	6.99	23.5	4.23 ^b^
58.6	7.47	1.99	0.74	1.58	7.11	23.6	4.81 ^ab^
88.6	7.36	1.97	0.77	1.64	7.04	23.8	4.93 ^a^
118.6	7.44	1.93	0.76	1.67	7.03	23.6	5.03 ^a^
148.6	7.41	1.95	0.78	1.64	7.10	23.6	5.21 ^a^
SEM	0.036	0.013	0.007	0.016	0.053	0.126	0.080
Probability							
Phytase	0.6474	0.2340	0.2957	0.5611	0.2898	0.4066	0.0486
Zinc	0.7886	0.4891	0.5462	0.3831	0.9388	0.9703	0.0009
Phytase vs. Zinc	0.1001	0.1090	0.4574	0.8582	0.9141	0.8061	0.9596

^a,b^ Means within the same column with different superscripts differ by Tukey test (*p* ≤ 0.05). ^1^ Ronozyme HiPhorius 40,000 FYT/g (Novozymes A/S, Bagsvaerd, Denmark). ^2^ Formulated at 28.6, 58.6, 88.6, 118.6, and 148.6 mg/kg by adding a laboratory-grade ZnSO_4_·7H_2_O; the analyzed values were 30.1 ± 0.73, 61.6 ± 0.13, 90.4 ± 1.60, 123.6 ± 1.99, and 151.9 ± 1.84 mg/kg. ^3^ Tibia Zn content at 28 d: Y = 3.771772048 + 0.018993389x − 0.000064899x^2^; R^2^ = 0.2083, *p* < 0.001.

**Table 4:** Liver zinc contents and organic markers from broiler chickens at 28 d of age fed diets with supplemental phytase and graded increases of Zn from 8 to 28 d ^1^.

	Liver Zn	Metallothionein	CuZnSOD	Myo-Inositol Concentration
Phytase, FYT/kg ^2^	mg/kg ^3,4^	(ng/mL) ^3,5^	(U/mL) ^6^	(µmol/dL)
None	88.0	6.79	10.86	140
4000	120.7	7.54	10.90	209
Zn, mg/kg ^2^				
28.6	94.1 ^b^	7.03	11.62 ^a^	180
58.6	104.0 ^ab^	7.11	11.54 ^a^	174
88.6	106.6 ^a^	7.15	11.17 ^ab^	174
118.6	107.3 ^a^	7.19	10.20 ^ab^	170
148.6	109.6 ^a^	7.34	9.89 ^b^	176
SEM	2.212	0.120	0.208	4.662
Probability				
Phytase	<0.0001	<0.0029	0.8621	<0.0001
Zinc	0.0005	0.9441	0.0206	0.9728
Phytase vs. Zinc	0.9142	0.9957	0.5698	0.9997

^a,b^ Means within the same column with different superscripts differ by Tukey test (*p* ≤ 0.05). ^1^ Formulated at 28.6, 58.6, 88.6, 118.6, and 148.6 mg/kg by adding a laboratory-grade ZnSO_4_·7H_2_O; the analyzed values were 30.1 ± 0.73, 61.6 ± 0.13, 90.4 ± 1.60, 123.6 ± 1.99, and 151.9 ± 1.84 mg/kg. ^2^ Ronozyme HiPhorius 40,000 FYT/g (Novozymes A/S, Bagsvaerd, Denmark). ^3^ Liver samples (n = 160 samples to Zn content, and 80 samples to metallothionein), plasma samples (n = 80), 8 replicates per treatment. ^4^ Liver Zn content at 28 d: Y = 85.43293704 − 0.35548959x − 0.00133237x2, R^2^ = 0.07, *p* < 0.05; X = dietary Zn content. ^5^ Contents from sample obtained from livers at 7 d were 4.01 ± 1.42 ng/mL. ^6^ Copper-zinc superoxide dismutase at 28 d: Y = 12.33221 − 0.01573x, R^2^ = 0.14, *p* < 0.001; X = dietary Zn content.

**Table 5:** Apparent ileal digestibility and total tract retention responses of broilers fed diets with supplemental phytase and graded increases of Zn from 8 to 28 d, dry matter basis ^1^.

	Apparent Ileal Digestibility ^3^					Zn Excreta ^4^					AME, kcal/kg
Phytase, FYT/kg ^2^	DM, %	IDEC, %	IDE, kcal/kg	CP, %	Zn, %	DM %	Intake mg/bird	Excretion mg/bird	Retention, mg/bird	Retention %	
None	67.8	70.9	3196	82.8	12.4	63.7	28.01	12.53	15.48	55.3	3115
4000	70.9	72.5	3264	84.7	24.6	66.0	27.65	10.25	17.40	62.9	3134
Zn, mg/kg ^3^											
28.6	69.7	72.3	3268	84.4	13.3	64.7	9.86 ^e^	3.86 ^e^	6.00 ^e^	60.9	3131
58.6	69.6	72.1	3241	84.2	21.5	65.0	18.58 ^d^	7.29 ^d^	11.29 ^d^	60.8	3126
88.6	69.4	71.7	3216	83.9	20.3	65.4	27.17 ^c^	10.87 ^c^	16.32 ^c^	60.1	3123
118.6	69.8	71.3	3219	83.7	19.4	65.6	37.51 ^b^	15.58 ^b^	21.94 ^b^	58.5	3119
148.6	68.2	71.2	3207	82.6	17.9	64.5	46.03 ^a^	19.35 ^a^	26.65 ^a^	57.9	3125
SEM	0.279	0.239	10.438	0.247	1.272	0.196	1.487	0.670	0.871	0.663	4.740
Probability											
Phytase	<0.0001	<0.0005	<0.0009	<0.0001	<0.0001	<0.0001	0.5860	<0.0001	<0.0005	<0.0001	0.0438
Zinc	0.0955	0.4512	0.3139	0.1349	0.0948	0.2134	<0.0001	<0.0001	<0.0001	0.1864	0.9577
Phytase*Zinc	0.1763	0.8305	0.7679	0.9564	0.0686	0.0702	0.6898	0.3641	0.5515	0.7342	0.9839

^a–e^ Means within the same column with different superscripts differ by Tukey test (*p* ≤ 0.05). ^1^ DM, dry matter; IDCE, ileal digestible energy coefficient; IDE, ileal digestible energy. ^2^ Ronozyme HiPhorius 40,000 FYT/g (Novozymes A/S, Bagsvaerd, Denmark). ^3^ Formulated at 28.6, 58.6, 88.6, 118.6, and 148.6 mg/kg by adding a laboratory-grade ZnSO_4_·7H_2_O; the analyzed values were 30.1 ± 0.73, 61.6 ± 0.13, 90.4 ± 1.60, 123.6 ± 1.99, and 151.9 ± 1.84 mg/kg. ^4^ Excretion and retention of Zn (mg/kg), respectively: Y = −0.37247 + 0.12858x, R^2^ = 0.87, *p* < 0.001; Y = 0.87236 + 0.17010x, R^2^ = 0.90, *p* < 0.001; X = dietary Zn content.

## Data Availability

The datasets generated and/or analyzed during the current study are available from the corresponding author upon reasonable request.

## References

[1] McCall K.A., Huang C., Fierke C.A. (2000). Function and Mechanism of Zinc Metalloenzymes. J. Nutr..

[2] Raulin J. (1869). Etudes clinques sur la vegetation. Ann. Sci. Nat. Bot..

[3] Vallee B.L., Bertini I., Gary H.B. (1986). A Synopsis of Zinc Biology and Pathology in Zinc Enzymes. Zinc Enzymes.

[4] McCance R.A., Widdowson E.M. (1942). The Absorption and Excretion of Zinc. Biochem. J..

[5] Oestreicher P., Cousins R.J. (1989). Zinc Uptake by Basolateral Membrane Vesicles from Rat Small Intestine. J. Nutr..

[6] Tacnet F.D., Watkins W., Ripoche P. (1990). Studies of Zinc Transport into Brush-Border Membrane Vesicles Isolated from Pig Small Intestine. Biochim. Biophys. Acta.

[7] Yu Y., Lu L., Luo X.G., Liu B. (2008). Kinetics of Zinc Absorption by In Situ Ligated Intestinal Loops of Broilers Involved in Zinc Transporters. Poult. Sci..

[8] Cherian M.G., Goyer R.A. (1978). Metallothioneins and Their Role in the Metabolism and Toxicity of Metals. Life Sci..

[9] Davis S.R., Cousins R.J. (2000). Metallothionein Expression in Animals: A Physiological Perspective on Function. J. Nutr..

[10] NRC-National Research Council (1994). Nutrient Requirements of Poultry.

[11] Underwood E.J., Suttle N.F. (1999). Zinc. The Mineral Nutrition of Livestock.

[12] Sandström B., Lönnerdal B., Mills C.F. (1989). Promoters and Antagonists of Zinc Absorption. Zinc in Human Biology.

[13] Angel R., Tamim N.M., Applegate T.J., Dhandu A.S., Ellestad L.E. (2002). Phytic Acid Chemistry: Influence on Phytin-Phosphorus Availability and Phytase Efficacy. J. Appl. Poult. Res..

[14] Eeckhout W., De Paepe M. (1994). Total Phosphorus, Phytate-Phosphorus and Phytase Activity in Plant Feedstuffs. Anim. Feed Sci. Technol..

[15] Rostagno H.S., Albino L.F.T., Calderano A.A., Hannas M.I., Sakomura N.K., Perazzo F.G., Rocha G.C., Saraiva A., Abreu M.L.T., Genova J.L. (2024). Brazilian Tables for Poultry and Swine: Composition of Foods and Nutritional Requirements.

[16] Cowieson A.J., Ruckebusch J.P., Sorbara J.O.B., Wilson J.W., Guggenbuhl P., Roos F.F. (2017). A Systematic View on the Effect of Phytase on Ileal Amino Acid Digestibility in Broilers. Anim. Feed Sci. Technol..

[17] Feijo J.C., Vieira S.L., Horn R.M., Altevogt W.E., Tormes G. (2023). Iron Requirements of Broiler Chickens as Affected by Supplemental Phytase. J. Anim. Sci..

[18] Soster P., Vieira S.L., Feijo J.C., Altevogt W.E., Tormes G.B. (2023). Dietary Phytase Effects on Copper Requirements of Broilers. Front. Vet. Sci..

[19] Young R.J., Edwards H.M., Gillis M.B. (1958). Studies on Zinc in Poultry Nutrition: 2. Zinc Requirement and Deficiency Symptoms of Chicks. Poult. Sci..

[20] Fundación Española para el Desarrollo de la Nutrición Animal (2018). Necesidades Nutriticionales Para Avicultura: Normas FEDNA.

[21] Cobb-Vantress (2022). Cobb500 Broiler Performance & Nutrition Supplement.

[22] Aviagen (2022). Ross 308, Ross 308 FF. Performance Objectives.

[23] EFSA FEEDAP Panel (EFSA Panel on Additives and Products or Substances Used in Animal Feed) (2014). Scientific Opinion on the Potential Reduction of the Currently Authorised Maximum Zinc Content in Complete Feed. EFSA J..

[24] Engelen A.J., Van der Heeft F.C., Randsdorp P.H.G., Smit E.L.C. (1994). Simple and Rapid Determination of Phytase Activity. J. AOAC Int..

[25] AOAC-International (2006). Official Methods of Analysis.

[26] Vogtmann H., Pfirter H.P., Prabuck A.L. (1975). A New Method of Determining Metabolisability of Energy and Digestibility of Fatty Acids in Broiler Diets. Br. Poult. Sci..

[27] Choct M., Annison G. (1992). Anti-Nutritive Effect of Wheat Pentosans in Broiler Chickens: Roles of Viscosity and Gut Microflora. Br. Poult. Sci..

[28] Kong C., Adeola O. (2014). Evaluation of Amino Acid and Energy Utilization in Dietstuff for Swine and Poultry Diets. Asian-Australas. J. Anim. Sci..

[29] Leung K.Y., Mills K., Burren K.A., Copp A.J., Greene N.D.E. (2011). Quantitative Analysis of Myo-Inositol in Urine, Blood, and Nutritional Supplements by High-Performance Liquid Chromatography Tandem Mass Spectrometry. J. Chromatogr. B Biomed. Appl..

[30] Gao R., Yuan Z., Zhao Z., Gao X. (1998). Mechanism of Pyrogallol Autoxidation and Determination of Superoxide Dismutase Enzyme Activity. Bioelectrochem. Bioenerg..

[31] AOAC-International (1995). Official Methods of Analysis.

[32] Shapiro S.S., Wilk M.B. (1965). An Analysis of Variance Test for Normality (Complete Samples). Biometrikam.

[33] Levene H., Olkin I. (1960). Robust Tests for the Equality of Variance. Contributions to Probability and Statistics.

[34] SAS Institute Inc (2012). SAS User’s Guide: Statistics.

[35] Tukey J.W. (1991). The Philosophy of Multiple Comparisons. Stat. Sci..

[36] López-Gambero A.J., Sanjuan C., Castro P.J.S., Suárez J., Fonseca F.R. (2020). The Biomedical Uses of Inositols: A Nutraceutical Approach to Metabolic Dysfunction in Aging and Neurodegenerative Diseases. Biomedicines.

[37] Aksorn S., Kanokkantapong V., Polprasert C., Noophan P., Khanal S.K., Wongkiew S. (2022). Effects of Cu and Zn Contamination on Chicken Manure-Based Bioponics: Nitrogen Recovery, Bioaccumulation, Microbial Community, and Health Risk Assessment. J. Environ. Manag..

[38] Pereira N.M.Z., Ernani P.B., Sangoi L. (2007). Disponibilidade de Zinco para o Milho Afetada pela Adição de Zn e pelo pH do Solo. Rev. Bras. Milho Sorgo.

[39] Addison T., Ghoshray A. (2023). Discerning Trends in International Metal Prices in the Presence of Nonstationary Volatility. Resour. Energy Econ..

[40] Philippi H., Sommerfeld V., Olukosi O.A., Windisch W., Monteiro A., Rodehutscord M. (2023). Effect of dietary zinc source, zinc concentration, and exogenous phytase on intestinal phytate degradation products, bone mineralization, and zinc status of broiler chickens. Poult. Sci..

[41] Valente J.D.T., Genova J.L., Kim S.W., Saraiva A., Rocha G.C. (2024). Carbohydrases and Phytase in Poultry and Pig Nutrition: A Review beyond the Nutrients and Energy Matrix. Animals.

[42] Chang L., Chiang S.H., Saltiel A.R. (2004). Insulin signaling and the regulation of glucose transport. Mol. Med..

[43] Cowieson A.J., Aureli R., Guggenbuhl P., Fru-Nji F. (2015). Possible Involvement of Myo-Inositol in the Physiological Response of Broilers to High Doses of Microbial Phytase. Anim. Prod. Sci..

[44] Bassi L.S., Teixeira L.V., Sens R.F., Almeida L., Zavelinski V.A.B., Maiorka A. (2021). High Doses of Phytase on Growth Performance, Bone Mineralization, Diet Utilization, and Plasmatic Myo-Inositol of Turkey Poults. Poult. Sci..

[45] Sens R.F., Bassi L.S., Almeida L.M., Rosso D.F., Teixeira L.V., Maiorka A. (2021). Effect of Different Doses of Phytase and Protein Content of Soybean Meal on Growth Performance, Nutrient Digestibility, and Bone Characteristics of Broilers. Poult. Sci..

[46] Bassi L.S., Teixeira L.V., Sens R.F., Sonálio K.C., Santos M.C., Kuritza L.N., Maiorka A. (2022). Effect of Phytase Supplementation and Drinking Water pH for Turkey Poults. Livest. Sci..

[47] Onosaka S., Cherian M.G. (1981). The Induced Synthesis of Metallothionein in Various Tissues of Rat in Response to Metals. I. Effect of Repeated Injection of Cadmium Salts. Toxicology.

[48] Searle P.F., Davison B.L., Stuart G.W., Wilkie T.M., Norstedt G., Palmiter R.D. (1984). Regulation, Linkage, and Sequence of Mouse Metallothionein I and II Genes. Mol. Cell. Biol..

[49] Karin M., Slater E.P., Herschman H.R. (1981). Regulation of Metallothionein Synthesis in HeLa Cells by Heavy Metals and Glucocorticoids. J. Cell. Physiol..

[50] Noor R., Mittal S., Iqbal J. (2002). Superoxide Dismutase: Applications and Relevance to Human Disease. Med. Sci. Monit..

[51] Potter S.Z., Zhu H., Shaw B.F., Rodriguez J.A., Doucette P.A., Sohn S.H., Durazo A., Faull K.F., Gralla E.B., Nersissian A.M. (2007). Binding of a Single Zinc Ion to One Subunit of Copper-Zinc Superoxide Dismutase Apoprotein Substantially Influences the Structure and Stability of the Entire Homodimeric Protein. J. Am. Chem. Soc..

[52] Reeves P.G., Briske-Anderson M., Johnson L. (1998). Physiologic Concentrations of Zinc Affect the Kinetics of Copper Uptake and Transport in the Human Intestinal Cell Model, Caco-2. J. Nutr..

[53] Ogiso T., Ogawa N., Miura T. (1979). Inhibitory Effect of High Dietary Zinc on Copper Absorption in Rats. II. Binding of Copper and Zinc to Cytosol Proteins in the Intestinal Mucosa. Chem. Pharm. Bull..

[54] Samman S. (1993). Dietary versus Cellular Zinc: The Antioxidant Paradox. Free Radic. Biol. Med..

[55] Liao X., Ang L., Lu L., Li S., Liu S., Zhang L., Luo X. (2022). Effects of Graded Levels of Zinc Oxide with an Organic Source of Zinc on Growth Performance, Carcass Traits, Meat Quality, and Zinc Status in Broilers. Biol. Trace Elem. Res..

[56] Zhang T.Y., Liu L.L., Zhang J.L., Zhang N., Yang X., Qu H.X., Xi L., Han J.C. (2018). Effects of Dietary Zinc Levels on the Growth Performance, Organ Zinc Content, and Zinc Retention in Broiler Chickens. Rev. Bras. Cienc. Avic..

[57] Chen X., He C., Zhang K., Wang J., Ding X., Zeng Q., Peng H., Bai J., Li L., Xuan Y. (2022). Comparison of Zinc Bioavailability in Zinc-Glycine and Zinc-Methionine Chelates for Broilers Fed with a Corn-Soybean Meal Diet. Front. Physiol..

[58] Foust R.D., Phillips M., Hull K., Yehorova D. (2018). Changes in Arsenic, Copper, Iron, Manganese, and Zinc Levels Resulting from the Application of Poultry Litter to Agricultural Soils. Toxics.

